# Role of dual PI3/Akt and mTOR inhibition in Waldenstrom's Macroglobulinemia

**DOI:** 10.18632/oncotarget.192

**Published:** 2010-11-17

**Authors:** Antonio Sacco, Aldo Roccaro, Irene M. Ghobrial

**Affiliations:** Dana-Farber Cancer Institute, Harvard Medical School, Boston, MA, USA

**Keywords:** malignancies, mTOR, PI3K, NVP-BEZ235, signal transduction

## Abstract

Tumorigenesis occurs due to synergistic interactions from a complex of signal transduction processes, including multiple onco-proteins and tumor suppressors such as Ras, Myc, PI3K/Akt/mTOR, Her-2/Neu, p53 and PTEN. Specifically, the PI3K/Akt and mTOR pathways have been shown to play a pivotal role on the initiation and progression of malignancies, enhancing cell survival by stimulating cell proliferation, and inhibiting apoptosis. Therefore, it is critical to examine therapeutic agents that explicitly target both the PI3K/Akt and mTOR signaling cascades in diseases, such as Waldenstrom Macroglobulinemia (WM), that harbor activation of the PI3K/Akt pathway. We demonstrated that dual targeting of the PI3K and mTOR pathways by the novel inhibitor NVP-BEZ235, exhibited toxicity on WM cells by directly targeting the tumor clone and indirectly through an effect on the bone marrow milieu. These findings suggest that dual targeting of the PI3K and mTOR pathways is a better modality of targeted therapy for tumors that harbor activation of the PI3K/mTOR pathways, such as in WM.

## INTRODUCTION

Waldenstrom Macroglobulinemia (WM) is a rare, low-grade, IgM secreting, lymphoplasmacytic lymphoma, characterized by the presence of lymphoplasmacytic cells in the bone marrow and IgM secretion in the peripheral blood. We have recently demonstrated that primary WM cells present with a constitutive activation of the PI3K/Akt pathway, sustained by decreased expression of PTEN at the gene and protein levels, together with constitutive activation of Akt and mTOR, PI3K-downstream signaling cascades [1]. It has been clearly demonstrated that PI3K pathway plays a pivotal role on the initiation and progression of malignancies, enhancing cell survival by stimulating cell proliferation and inhibiting apoptosis [2-6]. Signaling begins with the activation of receptor tyrosine kinases (RTKs). Upon activation by a ligand, RTKs engage and activate PI3K, which in turn converts membrane-bound phosphatidylinositol (4,5)-bisphosphonate (PIP2) to phosphatidylinositol (3,4,5)-triphosphonate (PIP3). PIP3 then activates Akt by phosphorylation [7,8]. Akt acts to promote cell proliferation and survival, and regulates multiple signaling pathways that maintain cell cycle, proliferation, and resistance to apoptosis such as BAD, caspases, IKK, GSK3, Forkhead-related transcription factor 1 (FKHR1), eNOS, and mTOR [4,7,8]. The mammalian target of rapamycin (mTOR) kinase leads to cell growth and proliferation [9]. mTOR exists in two distinct functional complexes, mTORC1 and mTORC2. mTORC1 (rapamycin sensitive) consists of mTOR and Raptor; its activation results in phosphorylation of p70S6 and 4E-BP1. mTORC2 consists of mTOR and the rapamycin-insensitive companion of mTOR (Rictor), and it results in Akt phosphorylation [10-13].

We have previously shown that targeting mTOR leads to significant clinical activity in these patients with up to 45% having partial remission when treated with a TORC1 inhibitor (RAD001, Novartis, NJ) [14]. However, patients did not have a complete remission, which indicates a mechanism of resistance to TORC1 exists in WM. Subsequently, we inquired as to whether dual inhibition of PI3K/Akt and mTOR pathways could potentially show higher cytotoxic activity in WM cells compared to PI3K or mTOR inhibitors alone, and found that dual targeting of the PI3K and mTOR pathways by the novel inhibitor NVP-BEZ235 exhibited higher cytotoxicity on WM cells compared to inhibition of the PI3K or mTOR pathways alone.

## RATIONAL FOR TESTING THE DUAL PI3K/AKT AND MTOR INHIBITOR IN WM CELLS

Reduced expression of PTEN appear to be one of the different genetic aberrations that leads to PI3K/Akt activation, responsible for fundamental cellular processes linked to tumorigenesis, such as cell-cycle regulation, cell proliferation and survival, cell adhesion and migration, and angiogenesis [7,8,15,16]. We identified that the expression of PTEN at gene level and protein level is lower in WM patients compared to control CD19+ cells. PTEN inactivating mutation or altered methylation status have not been described in WM, suggesting the possible role of epigenetics in silencing PTEN expression. For example, low PTEN levels could result from the de-regualted expression of microRNAs (miRNAs). miRNAs constitute a class of small, non-coding, 18–24 nucleotide RNAs, described for the first time in the nematode *Caenorabditis elegans* [17]. To date, more than 300 miRNAs have been discovered in humans [18]. miRNAs act as negative regulators of gene expression by binding to the 3′ untranslated region (UTR) of the target mRNAs with partial sequence complementarity and leading to translational repression [19]. By repressing several target mRNAs, mature miRNAs play a pivotal role in regulating development, cell differentiation, apoptosis, and cell proliferation [20]. We have previously performed miRNA profiling in WM [21], and found that miRNA-494 and -542-3p are over-expressed in WM patients as compared to the normal cellular counterpart, suggesting a possible role of miRNAs in silencing PTEN gene expression, since PTEN represents a predicted target for both miRNA-494 and -542-3p.

It is known that PTEN acts as negative regulator of Akt and mTOR [7], therefore, we subsequently confirmed that primary WM cells present with higher p-Akt and downstream p-mTOR protein levels compared to their normal cellular counterpart together with a higher expression of rictor and raptor, two different components of the protein kinase mTOR. These findings provide the biological preclinical evidence for testing a dual PI3K/Akt and mTOR inhibitor in tumors harboring constitutive activation of PI3K/Akt and mTOR signaling cascades, such as WM.

## DUAL INHIBITION OF PI3K AND MTOR PATHWAYS BETTER TARGET SIGNALING CASCADES IN WM CELLS AS COMPARED TO THE ACTIVITY EXERTED BY PI3K OR MTOR INHIBITORS USED AS SINGLE AGENTS

The efficacy of the dual NVP-BEZ235 in targeting Akt and mTOR pathways cells has been recently proven in WM cells, as well as in other IgM-secreting low-grade lymphoma cell lines, where the compound was able to specifically inhibit phosphorylation of Akt, and downstream GSK3_α/β_ and ribosomal protein S6 in a dose dependent manner in the WM cells, together with inhibited phosphorylation of mTOR, as well as of the downstream targets p70S6 and 4EBP1. In addition, inhibition of both Akt and mTOR kinase activities were also documented. mTOR represents a large protein kinase that exists as two different entities within cells: one that contains mTOR and raptor and another containing mTOR and rictor. The raptor-mTOR complex is sensitive to the mTOR inhibitor rapamycin, while the rictor-containing complex is rapamycin-insensitive [4-6] Notably, NVP-BEZ235 has been able to target both rictor and raptor in the context of mTORC1 and mTORC2 complexes indicating that this may result in down-regulating the rictor positive feedback loop on Akt activation [6]. Moreover while rapamycin inhibited raptor and did not target rictor, leading to phospho(p)-Akt up-regulation, NVP-BEZ235 induced significant p-Akt inhibition resulting from the dual targeting of both rictor and raptor. Interestingly, NVP-BEZ235 was equally or more effective in downregulating the mTOR-downstream targeted proteins p-p70S6 and p-4EBP1 compared to either PI3K or mTOR inhibitors when used alone.

## NVP-BEZ235-DEPENDENT INHIBITION OF AKT AND MTOR SIGNALING CASCADES LEADS TO TOXICITY IN WM CELLS, SUPPORTED BY INDUCTION OF APOPTOSIS AND CELL CYCLE ARREST IN TREATED CELLS

It is known that both PI3/Akt and mTOR pathways regulate cell growth and proliferation [22-25]; recent report indicates the efficacy of NVP-BEZ235 in inducing toxicity and reducing DNA synthesis in WM cells, supported by caspase-9, caspase-8, caspase-3, and PARP cleavage. Moreover, NVP-BEZ235 induced down-modulation of the anti-apoptotic protein Mcl-1, with an increased release of the second mitochondria-derived activator of caspases (Smac/DIABLO) from the mitochondria to the cytosol. In addition, inhibition of the inhibitor of apoptosis protein (c-IAP) was observed in WM cells exposed to dual inhibition of PI3K/Akt and mTOR pathways, based on the ability of Smac/DIABLO to abrogate the protective effects of IAPs [26]. In addition, NVP-BEZ235 has been shown to target forkhead box (FoxO) transcription factors, leading to cell cycle arrest in WM cells. Several reports indicate that FoxOs represent down-stream effectors of the PI3K/AKT pathway, and that phosphorylation of AKT leads to nuclear export and cytoplasm retention of phosphorylated FoxOs, with consequent inhibition of their transcriptional activity [27,28]. Notably, NVP-BEZ235-treated cells presented with inhibition of AKT-dependent p-FoxO1/O4/O3 expression; together with up-regulation of cell cycles inhibitors p27^kip1^ and p21^waf1^, leading to G1 cell cycle arrest and reduction of the S phase in WM cells exposed to the dual PI3K/Akt and mTOR inhibitor.

## DUAL INHIBITION OF PI3K/AKT AND MTOR PATHWAYS RESULTS IN TARGETING WM CELLS EVEN IN THE CONTEXT OF BONE MARROW MILIEU

**Figure 1:**
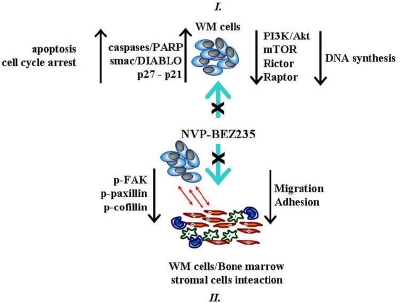
Dual inhibition of PI3/Akt and mTOR signaling cascades targets WM cells alone and WM cells in the context of bone marrow microenvironment

It is important to highlight the role of NVP-BEZ235 in targeting lymphoplasmacytic WM cells in the context of bone marrow (BM) microenvironment. BM milieu is represented by several cytotypes, including endothelial cells, fibroblasts, osteoblasts, osteoclasts, macrophages, and others that have been demonstrated to support tumor growth and to induce drug resistance in malignant cells [24,29]. Notably, the anti-tumor activity of NVP-BEZ235 against WM cells in the context of the BM milieu has been tested and validated both *in vivo* and *in vitro*. By co-culturing WM cells and primary BM stromal cells isolated from patients with WM, we have demonstrated that NVP-BEZ235 abrogated BMSC adhesion-induced phosphorylation of Akt and mTOR in WM cells, indicating that NVP-BEZ235 exerts its anti-tumor activity even when WM cells were in contact with the BM milieu. Importantly, dual inhibition of PI3K/Akt and mTOR pathways targeted rictor and raptor in WM cells, even when cultured in presence of BMSCs which induced up-regulation of raptor but not rictor. In addition, NVP-BEZ235 has been proven to inhibit adhesion and migration of WM cells to BMSCs, supported by inhibited phosphorylation of focal adhesion kinase, paxillin and cofilin, important proteins which act as key regulators of adhesion and cell migration. *In vivo* assays have been performed in order to better define and validate the anti-tumor activity of NVP-BEZ235 in the context of BM milieu and it has been demonstrated that treatment of WM cells with NVP-BEZ235 resulted in a significant inhibition of WM cells homing to the BM *in vivo*. A schematic representation of NVP-BEZ235 effects on WM cells is provided in Figure 1.

## CONCLUSION

Primary WM cells show constitutive activation of the PI3K/Akt and mTOR pathways, supported by decreased expression of PTEN. We have recently demonstrated that dual targeting of the PI3K and mTOR pathways by the novel inhibitor NVP-BEZ235 exhibited higher cytotoxicity on WM cells compared to inhibition of the PI3K or mTOR pathways alone, leading to WM cell toxicity, even when WM cells are cultured in the presence of BMSCs, both *in vitro* and *in vivo*. These studies therefore show that dual targeting of the PI3K and mTOR pathways is a better modality of targeted therapy for tumors that harbor activation of the PI3K/mTOR pathway, such as in WM.

## References

[1] Roccaro AM, Sacco A, Husu EN (2010). Dual targeting of the PI3K/Akt/mTOR pathway as an antitumor strategy in Waldenstrom macroglobulinemia. Blood.

[2] Pene F, Claessens YE, Muller O (2002). Role of the phosphatidylinositol 3-kinase/Akt and mTOR/P70S6-kinase pathways in the proliferation and apoptosis in multiple myeloma. Oncogene.

[3] Sande TVD, De Schrijver E, Heyns W, Verhoeven G, Swinnen JV (2002). Role of the phosphatidylinositol 3′-kinase/PTEN/Akt kinase pathway in the overexpression of fatty acid synthase in LNCaP prostate cancer cells. Cancer Res.

[4] Kim DH, Sarbassov DD, Ali SM (2002). mTOR interacts with raptor to form a nutrient-sensitive complex that signals to the cell growth machinery. Cell.

[5] Hara K, Maruki Y, Long X (2002). Raptor, a binding partner of target of rapamycin (TOR), mediates TOR action. Cell.

[6] Sarbassov DD, Ali SM, Kim DH (2004). Rictor, a novel binding partner of mTOR, defines a rapamycin-insensitive and raptor-independent pathway that regulates the cytoskeleton. Curr Biol.

[7] Sulis ML, Parsons R (2003). PTEN: from pathology to biology. Trends Cell Biol.

[8] Sansal I, Sellers W (2009). The biology and clinical relevance of the PTEN tumor suppressor pathway. J Clin Oncol.

[9] Hahn-Windgassen A, Nogueira V, Chen CC (2005). Akt activates mTOR by regulating cellular ATP and AMPK activity. J Biol Chem.

[10] Kim DH, Sarbassov DD, Ali SM (2002). mTOR interacts with raptor to form a nutrient-sensitive complex that signals to the cell growth machinery. Cell.

[11] Hara K, Maruki Y, Long X (2002). Raptor, a binding partner of target of rapamycin (TOR), mediates TOR action. Cell.

[12] Loewith R, Jacinto E, Wullschleger S (2002). Two TOR complexes, only one of which is rapamycin sensitive, have distinct roles in cell growth control. Mol Cell.

[13] Sarbassov DD, Ali SM, Kim DH (2004). Rictor, a novel binding partner of mTOR, defines a rapamycin-insensitive and raptor-independent pathway that regulates the cytoskeleton. Curr Biol.

[14] Ghobrial IM, Gertz M, LaPlant B (2009). A Phase II Trial of the Oral mTOR Inhibitor Everolimus (RAD001) in Relapsed or Refractory Waldenstrom's Macroglobulinemia. J Clin Oncol.

[15] Brader S, Eccles SA (2004). Phosphoinositide 3-kinase signalling pathways in tumor progression, invasion and angiogenesis. Tumori.

[16] Leleu X, Jia X, Runnel J (2007). The Akt pathway regulates survival and homing in Waldenstrom macroglobulinemia. Blood.

[17] Lee RC, Feinbaum RL, Ambros V (1993). The C-Elegans heterochronic gene Lin-4 encodes small RNAs with antisense complementarity to Lin-14. Cell.

[18] Bentwich I, Avniel A, Karov Y (2005). Identification of hundreds of conserved and nonconserved human microRNAs. Nat Genet.

[19] Xie X, Lu J, Kulbokas EJ (2005). Systematic discovery of regulatory motifs in human promoters and 3′UTRs by comparison of several mammals. Nature.

[20] He L, Hannon GJ (2004). MicroRNAs: small RNAs with a big role in gene regulation. Nat Rev Genet.

[21] Roccaro AM, Sacco A, Chen C (2009). microRNA expression in the biology, prognosis, and therapy of Waldenström macroglobulinemia. Blood.

[22] Vivanco I, Sawyers CL (2002). The phosphatidylinositol 3-Kinase AKT pathway in human cancer. Nat Rev Cancer.

[23] Pene F, Claessens YE, Muller O (2002). Role of the phosphatidylinositol 3-kinase/Akt and mTOR/P70S6-kinase pathways in the proliferation and apoptosis in multiple myeloma. Oncogene.

[24] Leleu X, Jia X, Runnel J (2007). The Akt pathway regulates survival and homing in Waldenstrom macroglobulinemia. Blood.

[25] Dancey JE (2004). Molecular targeting: PI3 kinase pathway. Ann Oncol.

[26] Tanimoto T, Tsuda H, Imazeki N (2005). Nuclear expression of cIAP-1, an apoptosis inhibiting protein, predicts lymph node metastasis and poor patient prognosis in head and neck squamous cell carcinomas. Cancer Lett.

[27] Ho KK, Myatt SS, Lam EW (2008). Many folks in the path: cycling with FoxO. Oncogene.

[28] You H, Pellegrini M, Tsuchihara K (2006). FOXO3a-dependent regulation of Puma in response to cytokine/growth factor withdrawal. J Exp Med.

[29] Mitsiades CS, Mitsiades NS, Munshi NC (2006). The role of the bone microenvironment in the pathophysiology and therapeutic management of multiple myeloma: interplay of growth factors, their receptors and stromal interactions. Eur J Cancer.

